# Complete genome sequence of *Thermodesulfovibrio* sp. strain TK500a, isolated from Nakabusa Hot Springs, Japan

**DOI:** 10.1128/mra.00756-25

**Published:** 2025-10-10

**Authors:** Toko Hisano, Shin Haruta

**Affiliations:** 1 Department of Biological Sciences, Tokyo Metropolitan University 12944, Hachioji, Tokyo, Japan; 2 Department of Biological Sciences, Purdue University 124080, West Lafayette, Indiana, USA; University of Southern California, Los Angeles, California, USA

**Keywords:** sulfate-reducing bacteria, terrestrial hot spring, *Nitrospirota*

## Abstract

Here we present the complete genome sequence of a sulfate-reducing bacterium in the phylum *Nitrospirota*, *Thermodesulfovibrio* sp. strain TK500a, newly isolated from Nakabusa Hot Springs, Nagano prefecture, Japan. The genome comprises a 2,018,434 bp chromosome with a guanine-cytosine content of 36.9% and 2,038 protein-coding sequences.

## ANNOUNCEMENT

Bacteria in the phylum *Nitrospirota* appear to have ecologically important roles in carbon, nitrogen, and sulfur cycling in terrestrial aquifers (1–3). It is of great interest to discuss the evolutionary traits of metabolic capabilities and ecological niches of this lineage (1, 4). Among them, the genus *Thermodesulfovibrio*, known as a thermophilic sulfate-reducing bacterium (5), is phylogenetically diverse, and elucidation of their physiological and genetic differentiation should provide important clues for understanding metabolic evolution (6–8).

A piece of microbial mats was collected at Nakabusa Hot Springs (36°23′20″N, 137°44′52″E, Nagano, Japan) as described previously (9), and was anaerobically cultivated. A pure culture of strain TK500a was obtained by three rounds of roll-tube method and dilution-to-extinction method, as reported previously (3), and grown at 70°C (see below). Here we report the whole genome sequence of strain TK500a to contribute to the evolutionary and physiological studies on *Nitrospirota*.

Strain TK500a was anaerobically grown at 70°C for 2 weeks in JCM479 medium (https://jcm.brc.riken.jp/) (pH 6.5) under N_2_:H_2_:CO_2_ (2:2:1, vol:vol:vol) atmosphere. Genomic DNA was extracted and purified using the Genomic Tip Kit (Qiagen) and sheared with Megaruptor 3 (Diagenode) to approximately 10–25 kb. A sequencing library was prepared using the SMRTbell Prep Kit 3.0 and the SMRTbell gDNA Sample Amplification Kit (PacBio). The library was sequenced with the Revio (PacBio). High-fidelity reads were obtained through adapter removal and *Q* ≥ 20 filtering using SMRT Link (v.13.1.0.221970, PacBio). Reads were trimmed using lima (v.2.9.0) (https://github.com/pacificbiosciences/barcoding), and PCR duplicates were removed using pbmarkdup (v.1.0.3) (https://github.com/PacificBiosciences/pbmarkdup). High-quality reads filtered using Filtlong (v.0.2.1) (https://github.com/rrwick/Filtlong) to remove reads of <1,000 bases were assembled using Flye (v.2.9.3) (10). A circularized assembly was confirmed with Bandage (v.0.8.1) (11), and assembly quality was assessed with CheckM2 (v.1.0.1) (12). Genome annotation was performed using the DFAST pipeline (13). Default parameters were used for all software analyses.

**Fig 1:**
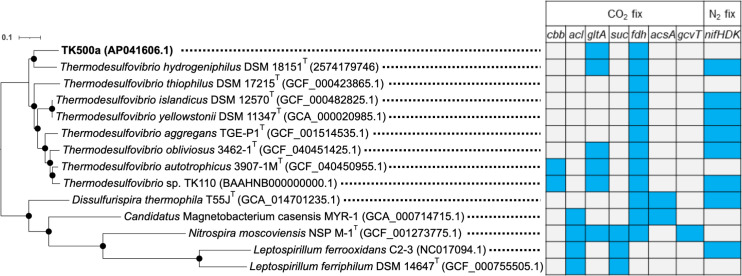
Phylogenetic classification of strain TK500a. Maximum likelihood phylogenetic tree was generated by ezTree (14) using 212 single-copy marker proteins. The scale bar represents the number of substitutions per site. Bootstrap values (1,000 replicates) were 100% for all nodes. The presence or absence of each homologous gene for CO_2_ fixation and N_2_ fixation is shown using color coding: blue for presence and white for absence. *cbb*, ribulose-1,5-bisphosphate carboxylase (Calvin-Benson-Bassham cycle); *acl*, ATP-citrate lyase (reductive tricarboxylic acid [TCA] cycle); *gltA*, *Si*-/*Re*-citrate synthase; *suc*, succinyl-CoA synthetase (oxidative TCA cycle/reversed oxidative TCA cycle); *fdh*, formate dehydrogenase (Wood-Ljungdahl pathway and reductive glycine pathway); *acsA*, CO dehydrogenase (Wood-Ljungdahl pathway); *gcvT*, glycine synthase (reductive glycine pathway); *nifHDK*, molybdenum-iron nitrogenase (N_2_ fixation).

The assembled genome of strain TK500a contained a single contig of 2.0 Mb with 36.9% guanine-cytosine (GC) content (Table 1). A molecular phylogenetic tree supported that strain TK500a was classified into the genus *Thermodesulfovibrio* (Fig. 1). The 16S rRNA gene sequence showed 96.9%, 96.7%, and 96.3% identity with those from *Thermodesulfovibrio obliviosus* (CP144374.1), *Thermodesulfovibrio autotrophicus* (CP144373.1), and *Thermodesulfovibrio hydrogeniphilus* (2574179745), respectively, determined through the local alignment (https://www.ebi.ac.uk/jdispatcher/psa/emboss_water). Average nucleotide identity using BLAST (https://github.com/widdowquinn/pyani) of strain TK500a was 74%–77% with *Thermodesulfovibrio* sp. shown in the tree in Fig. 1.

**TABLE 1:** Assembly and genomic features of *Thermodesulfovibrio* sp. strain TK500a

Features	Strain TK500a
Genome size (bp)	2,018,434
High-fidelity reads	37,522
Raw read N_50_ (bp)	7,251
Genome coverage	123
Number of contigs	1
Completeness (%)	100
Contamination (%)	0
GC content (%)	36.9
Coding sequence	2,038
rRNAs	3
tRNAs	47

Carbon and nitrogen fixation-related genes were explored (Fig. 1). Strain TK500a does not possess any key genes for the Calvin-Benson-Bassham cycle and the reductive tricarboxylic acid (TCA) cycle. No homologous genes encoding CO dehydrogenase and glycine synthase were found in the genome of strain TK500a, as reported for other *Thermodesulfovibrio* sp. (2, 3). Some of the genes for the oxidative/reversed oxidative TCA cycle (15, 16) were missing in *Thermodesulfovibrio*. N_2_-fixation genes were not detected from strain TK500a.

## Data Availability

The genome sequence has been deposited at DDBJ/ENA/GenBank under accession number AP041606. The associated BioProject and BioSample accession numbers are PRJDB20001 and SAMD00879868, respectively. Raw sequence reads are available in the DDBJ Sequence Read Archive under accession number DRR635009.
